# Photodynamic Therapy as an Alternative Therapeutic Tool in Functionally Inoperable Oral and Oropharyngeal Carcinoma: A Single Tertiary Center Retrospective Cohort Analysis

**DOI:** 10.3389/fonc.2021.626394

**Published:** 2021-03-04

**Authors:** Arnaud Lambert, Lotte Nees, Sandra Nuyts, Paul Clement, Jeroen Meulemans, Pierre Delaere, Vincent Vander Poorten

**Affiliations:** ^1^Department of Otorhinolaryngology, Head and Neck Surgery, University Hospitals Leuven, Leuven, Belgium; ^2^Department of Oncology—Section Head and Neck Oncology, KU Leuven, Leuven, Belgium; ^3^Department of Oncology—Section Experimental Radiotherapy, KU Leuven, Leuven, Belgium; ^4^Radiation Oncology, University Hospitals Leuven, Leuven, Belgium; ^5^Department of Oncology—Section Experimental Oncology, KU Leuven, Leuven, Belgium

**Keywords:** photodynamic therapy, oral, oropharyngeal, outcome, head and neck squamous cell carcinoma

## Abstract

**Background:** Head and neck cancer is typically treated with surgery, radiotherapy, chemoradiation, or a combination of these treatments. This study aims to retrospectively analyse oncological outcomes, adverse events and toxicity of treatment with temoporfin-mediated photodynamic therapy at a single tertiary referral center. More specifically, in a selected group of patients with otherwise (functionally) inoperable oral or oropharyngeal head and neck squamous cell carcinoma.

**Methods:** Twenty-six consecutive patients who received photodynamic therapy for oral or oropharyngeal squamous cell carcinoma from January 2002 until July 2019 at the University Hospitals Leuven were included. These were (1) patients with an accessible recurrent or new primary tumor in an extensively treated area of the head and neck, not suitable for standard treatment, or (2) patients that were judged medically unfit to undergo standard treatment modalities.

**Results:** Complete tumor response immediately after PDT was obtained in 76.9% of cases. During follow-up, a proportion of CR patients did recur, to reach recurrence-free rates at six months, one year and two years of 60.6%, 48.5% and 32.3%. Local control at the PDT treated area was 42.3% with a median recurrence free interval time of 9 months. Recurrence-free interval was statistically more favorable for oropharyngeal squamous cell carcinoma (with or without oral cavity extension) in comparison to oral cavity squamous cell carcinoma alone (*p* < 0.001). During a median follow-up period of 27 months, we report new tumor activity in 80.8% of patients. Median overall and disease-specific survival time was 31 and 34 months, respectively. Most reported adverse events were pain after treatment and facial edema. At the end of follow-up, swallowing and upper airway functionality were preserved in 76.9 and 95.7% of patients, respectively.

**Conclusion:** Photodynamic therapy is a valuable treatment option in highly selected patients with oral and/or oropharyngeal (functionally) inoperable head and neck squamous cell carcinoma. Treatment with this alternative modality can induce durable local control in an important fraction of treated patients, with an acceptable toxicity profile.

## Introduction

Head and neck cancer is a major health problem with substantial morbidity and mortality. Worldwide, this is the sixth most common cancer (1) and the eight most common cause of cancer-related death (2). In Belgian men, it is the fourth most common cancer, with an incidence of 36.2 per 100,000 people in 2016. The median age of patients with oral cavity and pharyngeal cancer at diagnosis in Belgian patients is 63.7 years in men and 65.8 years in women. For newly diagnosed cases in Belgium from 2012 until 2016, the 5-year relative survival rate is 51.2 and 59.4% for men and women, respectively (3). Despite the progress in standard treatment, tumor recurrence and second primary tumors occur in many patients after extensive combined treatment, which poses the need for novel therapies.

In this study, we investigate the oncological outcome and adverse events (AE) of photodynamic therapy (PDT), as a mildly invasive treatment alternative for head and neck cancer. It combines a photosensitizing agent, oxygen and a light source of a specific activating wavelength to establish a cytotoxic effect (4). The intravenously injected photosensitizing agent concentrates preferably in tumor tissue as compared to adjacent healthy tissue because it is taken up by cells with an elevated metabolic rate that have, in addition, less potency to get rid of the drug by exocytosis compared to healthy cells. Approximately 4–5 days (96–120 h) following injection, the ratio of photosensitizer concentration in tumor as opposed to healthy tissue is maximal. Illumination is performed by a laser that is set to a specific wavelength in relation to the absorption characteristics of the administrated photosensitizer. The activation of the photosensitizer by the emitted photons during illumination, creates an excited state that can either emit fluorescence to lose excess energy or form a triplet state of the photosensitizer. Combined with oxygen, this activated state interacts with organic molecules producing free radicals that are cytotoxic and vasculotoxic, provoking apoptosis, and a local inflammatory response which can cause a systemic antitumor immunological reaction (5, 6). Consequently, it causes tumor necrosis and ischemia. The tissue neighboring the illuminated area is, in part, spared because of the significantly lower concentration of photosensitizer stored in healthy cells. As a result, its architecture and collagen framework is protected and this facilitates recovery (7). In the past, PDT was not used very widely because of the lack of an appropriate photosensitizer. Since the development of second-generation photosensitizers that have more depth of penetration and less side effects, there is an increasing interest in PDT (8). We use one of the most potent available second generation photosensitizers, meta-tetra(hydroxyphenyl)chlorin (mTHPC), which has a maximal absorption peak at 652 nm, necessitating a diode laser with exactly this wavelength (9).

Ablative surgery and radiation therapy with or without chemotherapy are the most commonly used therapeutic options in head and neck oncology. PDT offers an alternative treatment modality, in well-selected patients possibly providing local tissue preservation resulting in less post-treatment morbidity and a better functional outcome while maintaining adequate local tumor control. In contrast, ablative surgery in these instances can result in significant loss of function regarding speech and swallowing as well as a poor cosmetic outcome (5, 10). Radiotherapy, especially re-irradiation, while sparing the local anatomy, is at risk of rendering the tissue afunctional due to radiation induced fibrosis or necrosis (11). PDT, however, also has complications, such as pain, burns and edema of the tongue (5, 7).

In PDT, the photosensitizer can be activated by superficial illumination to a depth of 2–10 mm due to the physical properties of the used wavelength of light in combination with the tissue properties; as such it is effective only for superficial tumors. For tumors with a depth of more than 10 mm, interstitial PDT (iPDT), i.e., bypassing the issue of depth of tumor invasion by implanting the tumor with light sources (laser fibers) (5) can be an alternative. A systematic review by De Visscher et al. illustrated that PDT for head and neck squamous cell carcinomas (HNSCC) in the palliative setting enhanced the quality of life in patients with limited remaining treatment options. In a curative setting, they concluded that there were not enough data to support its use (12).

We conducted this retrospective cohort study in our tertiary care center to evaluate treatment with Temoporfin-mediated PDT for oral cavity and oropharyngeal cancer. This treatment option is offered to a small selected subgroup of patients with a history of head and neck cancer already treated with conventional therapeutic strategies, especially when a significant functional impairment is expected when treated with either salvage surgery or (re-)irradiation. Provided the tumor is locally accessible for illumination, our multidisciplinary tumor board typically advises the use of PDT as a means to offer a less invasive and less toxic alternative as opposed to major ablative surgery or (re-)irradiation, respectively. In this study, patient characteristics, outcome and reported adverse effects were investigated and subsequently compared to data in the literature.

## Materials and Methods

### Patients

In this retrospective cohort study, all consecutive patients with oral cavity and or oropharyngeal cancer treated with mTHPC photodynamic therapy at the University Hospitals Leuven from January 2002 until July 2019 were included. All PDT treatments were performed by one senior head and neck surgeon (Vander Poorten V.). Treatment with mTHPC was offered if the tumor board concluded no other conventional treatment option to be suitable other than palliative chemotherapy or immunotherapy. Patients were selected for this treatment in case of (1) local recurrence or a new primary, without evidence distant metastasis, following extensive previous treatments including surgery, radiation or chemoradiation, where salvage surgery or re-irradiation is not an option, (2) surgically unacceptable functional impairment [functional inoperability (13)] or (3) patient refusal or being medically unfit to undergo conventional treatments. The accessibility of the tumor for superficial illumination was a prerequisite for PDT treatment selection. In tumors more difficult to access with perpendicular superficial illumination, interstitial PDT was used. The study population consists of 26 patients (10 women, 16 men) with a median mean age of 59 years old. Data of all included patients were extracted from their electronic medical file. The patient characteristics including substance abuse risk factors are listed in Table 1.

**Table 1:** Patient characteristics: Age, gender, and risk factors for *n* = 26 patients, with oral cavity and/or oropharyngeal cancer treated with photodynamic therapy.

**Patient characteristics (*****n*** **=** **26)**		
Age at diagnosis of PDT tumor	Mean (SD)	61.09 (8,436)
**Gender**	***n*** **(%)**	
Male		16 (61.5)
Female		10 (38.5)
**Risk factors**	***n*** **(%)**	
Alcohol alone		3 (11.5)
Smoking alone		1 (3.8)
Alcohol + smoking		21 (80.8)
No abuse		1 (3.8)
**Primary tumor before PDT**	***n*** **(%)**	
Unknown primary		2 (7.7)
Oral cavity		6 (23.1)
Oropharynx		9 (34.6)
Hypopharynx		1 (3.8)
Larynx		4 (15.4)
Oral cavity and oropharynx		2 (7.7)
Hypopharynx and larynx		2 (7.7)
**Second primary tumor before PDT**	***n*** **(%)**	
Oral cavity		3 (11.5)
Oropharynx		3 (11.5)
**PDT tumor origin**	***n*** **(%)**	
New primary		12 (46.2)
First rec of first primary		7 (26.9)
Second rec of first prim		4 (15.4)
PDT tumor = first primary		1 (3.8)
First rec of second primary		2 (7.7)
**Sum of treatments before PDT**	***n*** **(%)**	
No treatment before PDT		1 (3.8)
Ablative surgery		2 (7.7)
Primary RT alone		6 (23.1)
Primary RCT alone		2 (7.7)
Ablative surgery + RT		14 (53.8)
Primary RCT + salvage surgery		1 (3.8)

*Rec, recurrence; RT, radiotherapy; RCT, radiochemotherapy*.

### Methods

**Figure 1:**
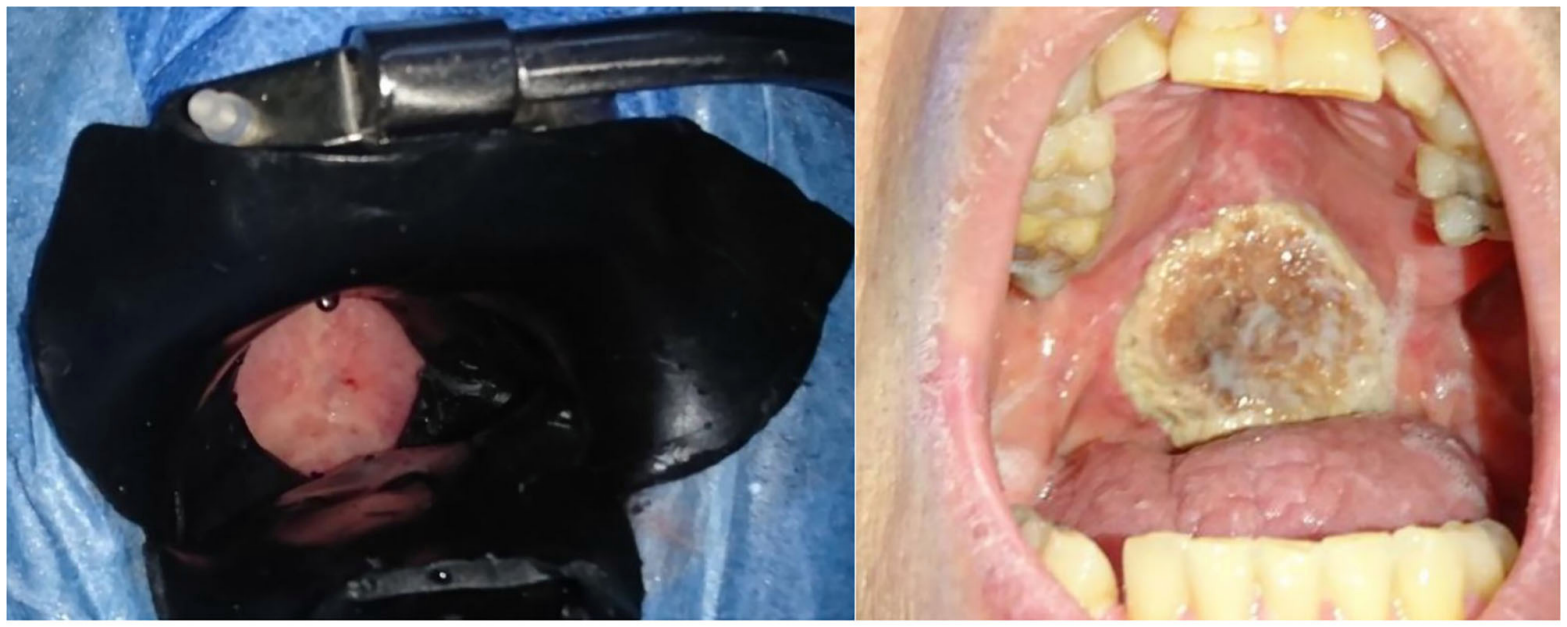
Left panel: Superficial Illumination of a tumor located on the soft palate (T2N0M0). Black shielding wax protects the surrounding tissue during illumination, including a margin of 1 cm. This patient had already received radiotherapy at this site. Right panel: tumor site necrosis of the tumor in the same patient 4 weeks after PDT.

Upon selection, the tumor location was specified and the tumor area and invasion depth were measured using high resolution CT scan, MRI scan or clinically if there was no evidence for the presence of a tumor on imaging or the measurements were not specified. A senior pathologist evaluated the histology. Previous treatments details for the index head and neck tumor (radiation, surgery, or chemotherapy) and smoking and alcohol consumption were extracted from the patient's medical file. The TNM classification (UICC 7th edition) was used to describe the locoregional anatomical extent of the tumor. Seventy-two to one-hundred and twenty hours (mean: 97.8 h, SD: 9.4) following intraveneous mTHPC administration, laser illumination was performed under general anesthesia using a Ceralas® 652 nm diode laser with microlens fiber (Biolitec, Jena, Germany). Taking into account a healthy tissue margin around the tumor of 5–10 mm, the remaining surrounding tissue was protected with black shielding wax (Figure 1). Patients were treated with superficial or/and interstitial PDT. During follow-up the treatment specific adverse events, swallowing and upper airway function, and the patients' alcohol use and smoking habits were recorded. Treatment modalities following the (first) PDT session were listed as well. All patients were repeatedly examined in the head and neck area to look for any tumor recurrence or other primary tumor, following a fixed follow-up protocol: 1 visit every 2 months the first 2 years, then every 3 months the 3rd year, every 4 months the 4th year, every 6 months the 5th year, and finally yearly until 10 years of follow-up. Baseline imaging using MRI (magnetic resonance imaging) was routinely obtained within a 2–6 months period for treatment response evaluation (14).

The primary outcomes of this study were:

1. tumor response to PDT, according to the RECIST1.1 (15) criteria: complete response (CR), partial response (PR) (≥30% reduction), stable disease (SD) (30% reduction – 20% increase) and progressive disease (PD) (≥20% increase). “Complete response” means complete resolution without clinical and radiological (MR/CT) evidence of tumor activity after PDT;
2. overall survival (time between the mTHPC injection and death or censoring/last follow-up), disease-free survival (time between mTHPC injection and first tumor recurrence/second primary tumor or last follow-up without disease) and disease-specific survival (time between mTHPC injection and death, patients that died from another cause being censored).

Secondary outcome was the occurrence of adverse events. Recorded adverse events were: pain, skin burns (due to inadvertent light exposure of the skin during the hypersensitive period following Temoporfin injection; graded and followed-up by our burns center), edema (as occurring in the entire head and neck region: facial, oral cavity, pharyngeal, and laryngeal edema). “Injection site reaction” was defined as every deviation from an uncomplicated injection at the site of mTHPC injection, including erythema during or after the administration, burns, pain, itch, hematoma, and swelling. Necrosis of the illuminated area is an expected consequence of PDT. This can be an adverse event if it is too extensive. Our policy is that every side effect is systematically noted in the electronic patient file. If there were no adverse events documented in the medical file, this means none had occurred. If any AE was documented more than 1 year after PDT, this was not assumed to be caused by PDT.

### Statistical Analysis

Statistical analysis was performed using IBM SPSS Statistics (version 27). Overall and disease-specific survival were assessed using the Kaplan-Meier method, as well as the recurrence-free interval. Univariate log-rank analysis was used to compare survival data between subgroups. An unpaired *T*-test was used to compare the means of two subgroups. Chi-square analysis was used to detect significant correlations between independent variables. Statistical significance was defined at the *p* < 0.05 level.

### Ethical Considerations

This study was carried out according to the prevailing ethical standards after obtaining approval by the Ethical Committee of the University Hospitals Leuven (approval number: MP010447).

## Results

### Patient Characteristics

Twenty-six patients received PDT in the University Hospitals Leuven. Two out of three patients were male (*n* = 16, 61.5%). The mean age at PDT was 61 years, with a minimum age of 47 and a maximum age of 79 years old. The vast majority of our patient population (*n* = 21, 80.8%) had a history of combined smoking and alcohol abuse. Three patients consumed alcohol alone. Of the remaining two patients, one was only a smoker and the other had no history in alcohol or smoking consumption whatsoever (Table 1). More than half (*n* = 14, 53.8%) of the patient population had undergone a combination of ablative surgery and radiotherapy before receiving PDT, six patients (23.1%) had a history of primary radiotherapy treatment, two patients (7.7%) received concurrent chemo- and radiotherapy before PDT and two patients (7.7%) had ablative surgery alone before PDT. One patient had a history of concurrent chemoradiation as well as ablative surgery before receiving PDT. Evidently, most of the patient population already had an extensive head and neck cancer history. In one patient PDT was selected as a suitable primary treatment modality: this patient had a large erythroplakia lesion with some invasive SCC spots located on the soft palate with extensions to the right retromolar trigone. This patient was very frail with a history of alcohol abuse, Korsakoff disease, and chronic kidney failure, making her unsuitable for ablative surgery or a full course of radiotherapy. The oncological and treatment history in terms of primary head and neck tumors before the oral cavity/oropharyngeal tumor treated with PDT is listed in Table 2, as well as an overview of recurrences before PDT treatment in Table 3. The mean time between the first primary tumor and the tumor treated with PDT varied from 7 to 348 months, with a median time of 66.5 months or 5.5 years.

**Table 2:** Tumor characteristics of primary head and neck SCC before diagnosis and treatment of the PDT tumor in 26 patients.

**Primary head and neck tumors before PDT tumor**			
	**First primary (*n* = 26)**	**Second primary (*n* = 6)**	**Third primary (*n* = 1)**
**Location**	***n*** **(%)**	***n*** **(%)**	***n*** **(%)**
Oral cavity (OC)	6 (23.1)	3 (11.5)	1 (3.8)
Oropharynx	9 (34.6)	3 (11.5)	
OC + oropharynx	2 (7.7)		
Hypopharynx	1 (3.8)		
Larynx	4 (15.4)		
Hypopharynx and larynx	2 (7.7)		
Unknown primary	2 (7.7)		
**TNM classification**	***n*** **(%)**	***n*** **(%)**	***n*** **(%)**
**clinical T class**			
cTx	2 (7.7)	1 (3.8)	
cT1	3 (11.5)		
cT2	4 (15.4)		
cT3	4 (15.4)		
cT4	3 (11.5)		
**Clinical N class**			
cN0	13 (50.0)	5 (19.2)	1 (3.8)
cN1	1 (3.8)		
cN2a	1 (3.8)		
cN2b	1 (3.8)		
**Pathological T class**			
pT1	4 (15.4)	4 (15.4)	
pT2	1 (3.8)		1 (3.8)
pT3	2 (7.7)		
pT4a	3 (11.5)		
**Pathological N class**			
pN0	1 (3.8)		
pN1	1 (3.8)		
pN2a	1 (3.8)		
pN2b	5 (19.2)		
pN3b	1 (3.8)		
Not available	0	1 (3.8%)	0
**Treatment**	***n*** **(%)**	***n*** **(%)**	***n*** **(%)**
RT + MRND	2 (7.7)		
Ablative surgery alone	3 (11.5)	5 (19.2)	1 (3.8%)
AS + ND	1 (3.8)		
AS + ND + adj. RT	7 (26.9)		
Primary RT	8 (30.8)		
Primary RCT	2 (7.7)		
RT + brachytherapy	2 (7.7)	1 (3.8)	
PDT	1 (3.8)		

*OC, Oral Cavity; RT, Radiotherapy; MRND, modified radical neck dissection; AS, Ablative surgery; ND, selective neck dissection; adj. RT, adjuvant radiotherapy; RCT, radiochemotherapy; PDT, photodynamic therapy*.

**Table 3:** Tumor characteristics of head and neck SCC recurrences before diagnosis and treatment of PDT tumor in 26 patients.

**Recurrences before PDT tumor**		
	**First recurrence (*n* = 9)**	**Second recurrence (*n* = 1)**
**Origin of recurrence**	***n*** **(%)**	***n*** **(%)**
First primary	8 (30.8)	1 (3.8)
Second primary	1 (3.8)	
No recurrence	17 (65.4)	25 (96.2)
**Location**	***n*** **(%)**	***n*** **(%)**
Oral cavity (OC)	6 (23.1)	1 (3.8)
Oropharynx	1 (3.8)	1 (3.8)
OC + oropharynx	1 (3.8)	
Larynx	1 (3.8)	
No recurrence	17 (65.4)	24 (92.3)
**TNM classification**	***n*** **(%)**	***n*** **(%)**
**Clinical T class**		
cT4a	1 (3.8)	
**Clinical N class**		
cN0	4	1 (3.8)
**Pathological T class**		
pT1	2 (7.7)	1 (3.8)
pT2	1 (3.8)	
pT3	1 (3.8)	
pT4a	1 (3.8)	
**Pathological N class**		
pN0	2 (7.7)	
*not available*	3 (11.5)	0
**Treatment**	***n*** **(%)**	***n*** **(%)**
Ablative surgery alone	3 (11.5)	1 (3.8)
AS + ND	4 (15.4)	
AS + adj. RT	1 (3.8)	
Primary RT	1 (3.8)	

*OC, Oral Cavity; AS, Ablative surgery; ND, selective neck dissection; adj. RT, adjuvant radiotherapy; RT, radiotherapy*.

### Tumor Characteristics

The most common anatomical site where PDT treatment was performed, was the oropharynx (*n* = 14, 53.8%), followed by the oral cavity (*n* = 8, 30.8%) and a combination of both locations (*n* = 4, 15.4%). Compared to the site of the first primary head and neck tumor in each patient, half of the tumors (*n* = 13, 50%) treated with PDT were located at a different site than their primary head and neck tumor; resulting in twelve patients (46.2%) with a new primary tumor and one patient where the tumor was in fact the first primary. The other half of tumors treated with PDT presented either as a first recurrence of the first primary (*n* = 7, 26.9%), a second recurrence of the first primary (*n* = 4, 15.4%) or a first recurrence of a second primary (*n* = 2, 7.7%). Of oropharyngeal anatomical subsites, the tumors treated with PDT were most frequently located on the soft palate (*n* = 10, 38.5%). Other subsites were more or less equally distributed between the lateral tonsillar wall or glossotonsillar sulcus (*n* = 2, 7.7%), the base of the tongue or vallecula (*n* = 3, 11.5) and the posterior pharyngeal wall (*n* = 3, 11.5%). The retromolar trigone or inner cheek area was mostly affected in the oral cavity (*n* = 8, 30.8%), usually in continuity with an adjacent subsite of the oral cavity (hard palate, lateral tongue) or oropharynx (soft palate). Only two cases presented as an isolated retromolar trigone tumor. Tumor characteristics and PDT treatment specifics per case are illustrated in Tables 4, 5 respectively.

**Table 4:** Tumor specific characteristics of 26 patients receiving photodynamic therapy for cancer of the oral cavity or oropharynx with oral cavity extension.

**PDT tumor characteristics**						
**n^**°**^**	**Location**	**L/R**	**Subsite**	**Rec. or new tumor**	**TNM**	**Tumor grade**
1	OC + OP	Left	Tonsillar fossa + trigonum retromolare	New primary	T2N0M0	NOS
2	OC + OP	Midline	Soft palate + hard palate	New primary	T2N0M0	Well-to medium diff
3	OP	Right	Base of tongue, vallecula	First rec. of first primary	T2N1M0	Poorly differentiated
4	OP	Midline	Base of tongue, vallecula	New primary	T1N1M0	Poorly differentiated
5	OP	Left	Tonsillar fossa, lateral wall	First rec. of first primary	TisN0M0	Carcinoma *in situ*
6	OP	Midline	Soft palate	First rec. of first primary	T1N0M0	NOS
7	OC	Midline	Hard palate	Second rec. of first primary	TisN0M0	Carcinoma *in situ*
8	OP	midline	Soft palate	First rec. of first primary	T2N0M0	NOS
9	OC + OP	right	Soft palate + trigonum retromolare	First primary	T1N0M0	NOS
10	OC	left	Tongue	First rec of second primary	T2N0M0	Poorly differentiated
11	OC	right	Trigonum retromolare, lateral tongue	New primary	T1N0M0	Well-to medium diff
12	OP	midline	Posterior oropharynx	New primary	T2N0M0	Well-differentiated
13	OP	right	Soft palate	First rec of second primary	T3N0M0	NOS
14	OC	right	Hard palate, trigonum retromolare	Second rec. of first primary	T1N0M0	Medium differentiation
15	OC	left	Hard palate, trigonum retromolare	Second rec. of first primary	T1N0M0	NOS
16	OC	right	Trigonum retromolare	New primary	T1N0M0	Well-to medium diff
17	OP	right	Soft palate	New primary	T2N1M0	NOS
18	OC + OP	Left	Soft palate + trigonum retromolare	New primary	T3N0M0	Well-to medium diff
19	OP	Right	Soft palate	New primary	T1N0M0	NOS
20	OP	Right	Posterior oropharynx wall	New primary	T1N0M0	NOS
21	OP	Right	Soft palate	New primary	TisN0M0	Carcinoma *in situ*
22	OP	Right	Base of tongue + floor of mouth	First rec. of first primary	T3N0M0	Well-differentiated
23	OC	Right	Floor of mouth	Second rec. of first primary	T3N0M0	Verrucous carcinoma
24	OP	Left	Soft palate, lateral wall	New primary	T2N0M0	Medium differentiation
25	OC	Left	Trigonum retromolare	First rec. of first primary	T1N0M0	Medium differentiation
26	OP	Right	Posterior oropharynx	First rec. of first primary	T2N0M0	Medium differentiation

*OC, oral cavity; OP, oropharynx; rec., recurrence; NOS, not otherwise specified; diff, differentiation*.

**Table 5:** Photodynamic treatment type and associated interventions for 26 patients receiving therapy for cancer of the oral cavity or oropharynx with oral cavity extension.

**PDT treatment characteristics**				
**n^**°**^**	**PDT type**	**Concomittant R/**	**Airway R/**	**Swallowing R/**
1	Surface illumination	None	None	None
2	Surface illumination	None	None	Planned NGT
3	Surface illumination	Selective ND	None	None
4	Surface illumination	lymph node excision	Planned tracheotomy	Planned NGT
5	Surface illumination	None	None	None
6	Surface illumination	None	None	None
7	Surface illumination	None	None	None
8	Surface illumination	None	None	None
9	Surface illumination	None	NONE	None
10	Surface illumination	None	Urgent tracheotomy	Unplanned NGT
11	Surface illumination	None	Pre-existing tracheostoma	None
12	Surface illumination	None	Planned tracheotomy	Planned PEG
13	Surface illumination	None	Pre-existing tracheostoma	None
14	Surface + interstitial ill.	None	None	None
15	Surface illumination	None	None	NGT on readmission
16	Interstitial PDT	None	None	None
17	Surface + interstitial ill.	MRND	Planned tracheotomy	Planned PEG
18	Surface illumination	None	Planned tracheotomy	Planned NGT
19	Surface illumination	None	None	None
20	Surface + interstitial ill.	None	Pre-existing tracheostoma	None
21	Surface illumination	None	Planned tracheotomy	Pre-existing PEJ
22	Interstitial PDT	None	Planned tracheotomy	Planned NGT
23	Surface illumination	None	Normal airway	None
24	Surface illumination	None	Normal airway	None
25	Surface + interstitial ill.	None	Normal airway	Planned NGT
26	Surface illumination	None	Planned tracheotomy	NGT on readmission

*NGT, nasogastric tube; PEG, percutaneous endoscopic gastrostomy; ill., illumination; MRND, modified radical neck dissection*.

### Treatment Characteristics

mTHPC was administered to the patients at a dose of 0.15 mg/kg. This resulted in a mean dose of 9.28 mg (median: 9.15 mg, range 5.55–12.90 mg) during a mean intravenous infusion time of 7.7 minutes (median: 8 min, range 5–10 min). The laser illumination had a mean total energy of 0.66 Watt per spot (median: 0.67 Watt, range 0.1–1.45 Watt). The superficially illuminated area consisted of one or more spots, with a mean spot size of 3 cm and a maximum spot size of 4 cm. If larger areas needed to be illuminated, several overlapping spots were used, with a mean of 3 spots (median: 3, range 1–4 spots). As a standard for superficial PDT every spot was illuminated for 200 s. Superficial PDT with surface illumination alone was performed in 76.9% (*n* = 20) of the patients, interstitial PDT in 7.7% (*n* = 2) and a combination of both surface and interstitial PDT in 15.4% (*n* = 4) of the patients. Of the 26 patients, two patients received interstitial PDT and four patients received a combination of interstitial PDT and superficial illumination. For interstitial PDT, minimum 4 and maximum 14 bare fibers were placed in the tumor tissue (mean: 9 fibers). The mean length of stay in the hospital was 17 days (median: 12.5 days, range 7–40 days). This includes pre-operative evaluation at the first date of admission, as well as any concomitant treatments performed before illumination (e.g., planned tracheotomy, neck dissection). After PDT illumination, the median length of stay in the hospital was 7 days (range 3–34 days). Follow-up ranged from 2 to 129 months, with a median time of 29.5 months. Out of our group of 26 patients 76.9% (*n* = 20) died during follow-up and six patients were alive at the end of follow-up. Median follow-up in these 6 patients is 22.5 months with a minimum of 3 months and a maximum of 62 months (median 22.5 months).

### Tumor Response

Complete response was obtained in 76.9% (*n* = 20) of the treated tumors at a mean follow-up time of 36.55 months (min: 2, max 129, and median 30.5 months). A partial tumor response was seen in 11.5% (*n* = 3) with a median follow-up time of 15 months. An equal amount of patients (11.5%, *n* = 3) presented with progressive disease within a median follow-up time of 6 months. Out of the 20 patients treated with surface illumination alone, 17 tumors showed complete response, one partial response and two tumors exhibited progressive disease. Four patients were treated with a combination of surface illumination and interstitial PDT, resulting in two complete and two partial responses. Finally, two patients were treated with interstitial PDT alone, showing complete response in one and progressive disease in the other. In Table 6, we provide an overview of the tumor response per PDT modality in relation to anatomical site subgroup (oral cavity alone vs. oropharynx ± oral cavity extension). Chi-square analysis shows a significant correlation favoring complete tumor response in the oropharynx subgroup (*p* = 0.03). No significant difference was found between superficial PDT, interstitial or combined PDT.

**Table 6:** Overview of tumor response according to RECIST criteria per subgroup and per PDT modality.

**Tumor response**							
**Subgroup**		OC alone (***n =*** 8)			OP & OP + OC extension (***n =*** 18)		
**PDT type**		**SI (*n* = 5)**	**iPDT (*n* = 1)**	**SI + iPDT (*n* = 2)**	**SI (*n* = 16)**	**iPDT (*n* = 1)**	**SI + iPDT (*n* = 2)**
Tumor Response	PD	1	0	0	1	1	0
	SD	0	0	0	0	0	0
	PR	1	0	2	0	0	0
	CR	3	1	0	14	0	2

*OC, oral cavity; OP, oropharynx; rec., recurrence; SI, surface illumination; iPDT, interstitial PDT; PD, progressive disease; SD, stable disease; PR, partial response; CR, complete response*.

### Recurrence

**Figure 2:**
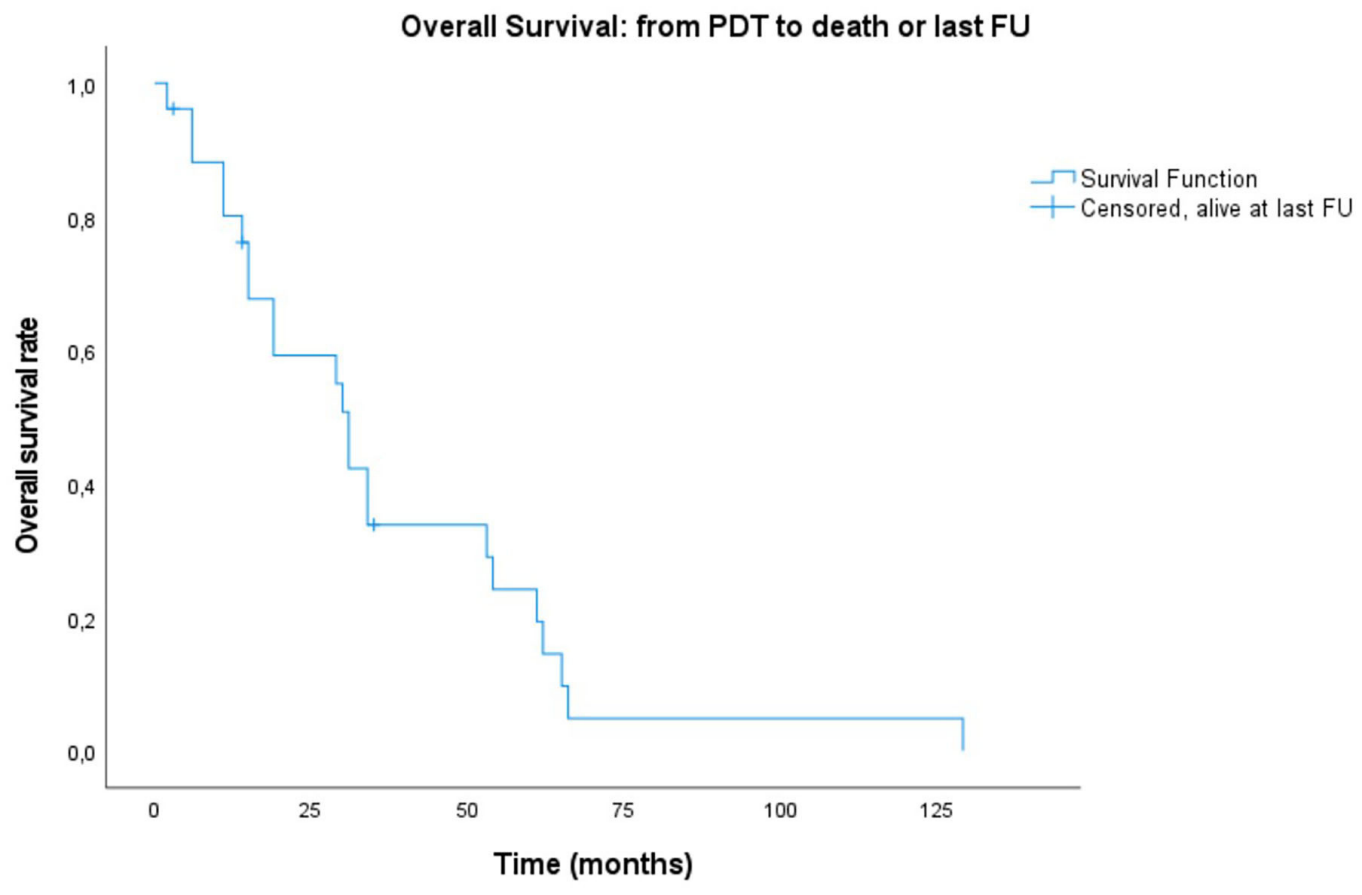
Kaplan–Meier plot showing overall survival of PDT patients, starting from illumination date to endpoint defined as either overall death or alive at last follow-up (FU).

Recurrence at the same site as the illumination area or at another site in the head and neck region (new primary tumor) occurred in the majority of the patients (80.8%, *n* = 21). In our case series 42.3% (*n* = 11) of tumors did not recur at the illuminated area. The median follow-up period of these patients was 27 months (range: 2–90; mean: 29.55 months). Consequently 57.7% (*n* = 15) did recur locally at a median follow-up time of 5 months (mean: 8.6 months). Overall recurrence, locally as well as at other sites in the head and neck area, occurred after a median time of 8 months, with a minimum of 22 days and a maximum of 90 months. The median follow-up time in the overall non-recurring patient group was 31 months (range: 2–52 months). About one third of our patients (*n* = 9, 34.6%) developed a separate primary tumor outside of the head and neck region in their follow-up period after PDT. Notably, the sites of these new primary tumors are also notoriously associated with alcohol abuse and smoking: esophageal cancer (*n* = 4), lung cancer (*n* = 3), breast cancer (*n* = 1), and bladder cancer (*n* = 1). Other separate primary tumors were prostate cancer and sarcoma. Six patients out of the 21 with a recurrence, developed this recurrence at a different or adjacent site in relation to the PDT treated area. This could be in the same subsite, but not in the illuminated tissue area, otherwise the recurrence was labeled as a local recurrence. Hence, the other 15 recurrences were identified as local recurrences, specifically at the level of the oropharynx (*n* = 8/15, 53.3%), the oral cavity (*n* = 5/15, 30%) or a combination of both (*n* = 2/15, 13.3%). In 90.5% (*n* = 19/21) of the patients who had head and neck tumor recurrence, local or otherwise, this was proven with a biopsy. In only two patients (*n* = 2/21, 9.5%), recurrence was based on clinical and radiological findings. Both these patients presented with a local recurrence in the previously illuminated site. Figure 2 shows a Kaplan–Meier plot of the recurrence-free interval after PDT. Two patients received more than one treatment with PDT. More specifically, one patient underwent a second (surface illumination) and third (interstitial) treatment with PDT because of incomplete response (oral cavity) and recurrence (oropharynx), respectively, with 5 and 7 months in between treatments, respectively. The second patient received a second treatment with surface illumination because of recurrence in the oropharynx, at another location as the first tumor treated with PDT 27 months earlier. After 6 months there was a new recurrence at the same site.

Median recurrence-free interval (RFI) is 9 months. Recurrence-free rates at 6 months, 1, and 2 years are 60.6, 48.5, and 32.3%. Five patients remained recurrence-free during the follow-up period. There was no statistically significant difference in RFI between tumors originating from a recurrence of an earlier primary in comparison to PDT tumors being a new primary (*p* = 0.209). A significantly worse RFI (*p* < 0.001) was observed in patients presenting with an oral cavity (OC) tumor alone, as opposed to those with an oropharyngeal tumor with or without oral cavity extension (OP ± OC). Median RFI was 3 months in the OC alone group and 22 months in the OP ± OC group (**Figure 4**).

### Survival

**Figure 3:**
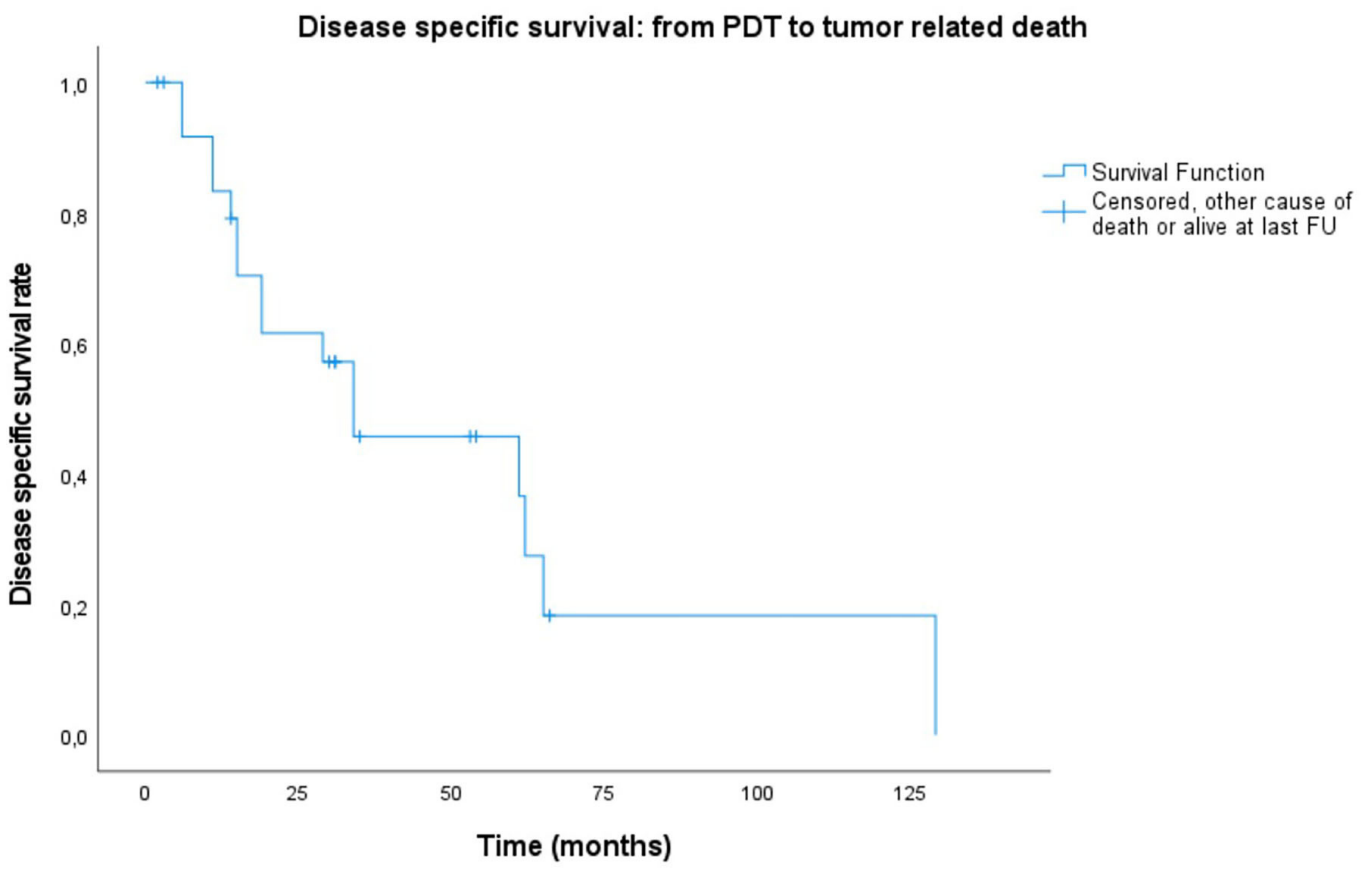
Kaplan–Meier plot showing disease-specific survival from PDT illumination date to endpoint defined as either tumor related death, other cause of death or alive at last follow-up (FU).

A lot of heterogeneity existed in whether or not the primary head and neck cancer was actually related with the tumor treated for PDT. Therefore, even though most patients were already under follow-up and care for a previous primary head and neck cancer, starting point for the survival plots was taken as the date of PDT illumination. Median overall survival (OS) of all treated patients was 31 months (mean: 36.6 months, range 2–129 months). Of 26 patients, three are alive to date and still in follow-up at 14, 46 and 47 months. Overall survival at 6 months, 1, and 2 years was 88.1, 80.1, and 59.2%, respectively. The 5-year overall survival was 24.2% (Figure 1). The majority of the patients died because of the tumor (*n* = 16, 61.5%), 26.9% (*n* = 7) died due to another cause (e.g., subdural hematoma, heart failure, other tumors, or unknown causes). The median disease-specific survival (DSS) was 34 months (mean: 51 months). Disease-specific survival at 6 months, 1, and 2 years was 91.7, 83.3, and 61.6%, respectively. The 5 year DSS was 36.6%. In comparing the PDT tumors regarded as a new primary in comparison to those being recurrences from earlier head and neck tumors, no statistically significant difference in survival was found (OS *p* = 0.209; DSS *p* = 0.907, and RFS *p* = 0.665). Similarly, there were no significant differences between the OC alone group and the OP ± OC when comparing OS (*p* = 0.399) and DSS plots (*p* = 0.210). Figures 2, 3 show the Kaplan–Meier plots on OS and DSS after PDT. Other factors such as age, gender, TNM classification, and substance abuse were examined but had no statistical significant impact on any survival plot. No significant difference was found in regards to PDT treatment type (superficial PDT vs. interstitial ± superficial PDT). Notably, complete tumor response as opposed to incomplete response (defined as partial response, stable disease, or progressive disease) showed no significant difference in terms of overall or disease specific survival.

### Adverse Events and Toxicity of Treatment

During hospitalization and follow-up, all adverse events (AE) were documented in the patient files. To get a clear view on PDT toxicity, we collected information on the frequency and duration of pain, swallowing impairment and the need for tube feeding, airway management (tracheostomy), treated area (tumor site) necrosis and duration of tissue healing, as well as any other local or systemic adverse event potentially linked to PDT.

All patients required a variable level of pain medication during and after treatment. Our department monitors pain daily with a visual analog pain scale (VAS 0–10) since 2010, so we only have scored data on pain during hospitalization in PDT patients treated after 2010 (*n* = 13, 50%). Median pain level at the day of treatment was a VAS score of 8 (*n* = 10, range: 0–10), at 1 day post-illumination the median pain level was VAS score of 5 (*n* = 13, range 0–7) and after 7 days we noted a median VAS score of 3 (*n* = 13, range 0–6). Lastly, to assess the need for pain medication after discharge we reviewed the documented pain medication intake 1 month post PDT. We divided the need for pain medication in the following categories: no need for pain medication, conventional oral medication only (paracetamol, NSAID), conventional oral pain medication in conjunction with oral opioid medication and lastly the necessity for transcutaneous continuous opioid pain medication. In five patient case files there was no mention of pain medication and this was regarded as missing data. At 1 month, a minority of patients was pain free without any medication (*n* = 7, 26.9%), over half of our patients (*n* = 14, 53.8%) had controlled pain levels using appropriate pain medication. Of these, five (35.7%) needed conventional pain medication only, three (21.4%) needed an additional oral opioid and six (42.9%) were dependent on continuous transcutaneous opioid medication for pain relief. After discharge we documented four readmissions (15.4%) within 1 month after discharge, three because of pain and dysphagia, one patient was admitted due to an aspiration pneumonia. One patient only reported inadequate pain relief and was discharged 2 days later. Another patient with mainly dysphagia received a reintroduction of nasogastric tube for feeding and was discharged with the nasogastric tube 6 days later. The last patient who was readmitted presented with pain and secondary dysphagia, after adjusting the pain medication discharge was possible at day 11.

Swallowing capacity of these patients is undeniably affected by PDT treatment in the short-term post-treatment period. Partially on its own due to tissue necrosis and loss of function to some degree, but also due to the associated pain. Over half of the patient population did not require any tube feeding (*n* = 15, 57.5%) in the hospital. Planned tube feeding was foreseen for the remaining eight patients. The majority of planned tube feeding was anticipated for the oropharyngeal tumors (*n* = 4 NGT, *n* = 2 PEG, *n* = 1 pre-existing PEJ) as opposed to the oral cavity tumors (*n* = 1 NGT). Five patients received a nasogastric tube at the time of hospitalization, two patients a planned percutaneous gastrostomy (PEG) tube, and one patient had a pre-existing percutaneous jejunostomy (PEJ) tube. The remaining three patients that required unplanned nasogastric tube feeding for dysphagia in the short term period were: two patients during readmission and one patient due to an unforeseen emergency tracheotomy and admittance to the ICU. Four out of the five patients with planned nasogastric tube feeding had their feeding tubes successfully removed by a median time of 13.5 days (range: 5–8 days, mean 12.5). Unfortunately two patients out of those four needed their nasogastric tube reintroduced within the short term follow-up period of 1 month. One patient out of the total five could not be accounted for, regarding timing of tube removal, due to transfer to another center for revalidation purposes. Standardized swallowing scores were not routinely used to assess swallowing capabilities outside of prospective controlled studies, as this is a retrospective case study we have no standardized evaluation data to report. However, clinically at the end of a median follow-up time of 29.5 months, the majority of patients (*n* = 18, 69.2%) had full oral intake. In those remaining eight who did eventually receive a PEG/PEJ tube (either planned or otherwise), two patients had their PEG tube successfully removed, two patients were co-dependent on tube feeding and oral feeding, three patients had hardly any oral feeding capability and mainly relied on tube feeding. Finally, one patient was entirely dependent on tube feeding due to aspiration risk. Thus, a clinically functional and preserved swallowing function could be observed for 76.9% (*n* = 20/26) of cases. Partially preserved swallowing function was present in 7.7% (*n* = 2/26) and dysfunctional swallowing with persistent dysphagia was present in 11.5% (*n* = 3/26) of patients. Only one patient (3.8%) showed complete loss of swallowing function due to treatment and was completely dependent on PEG tube.

The overall majority of patients was treated with PDT without a planned tracheotomy (*n* = 15, 57.7%). Three patients already had a permanent tracheostoma due to a laryngectomy in the past (*n* = 3, 11.5%). Most tracheostomies (*n* = 7, 26.9%) were planned and performed in the same hospitalization as the PDT treatment, before mTHPC injection and illumination. All planned tracheostomies were performed for patients that had PDT in the oropharynx. Namely, two out of three base of tongue carcinomas and three out of eight soft palate tumors. All (*n* = 3) posterior oropharynx tumors had airway management: two received a planned tracheostomy and one had a pre-existing tracheostomy. In only one case an urgent unplanned bedside tracheotomy under local anesthesia was necessary, 2 days after PDT illumination, due to laryngeal edema following prolonged illumination during difficult fiberoptic intubation. Notably this patient in particular had a history of a hemi-laryngectomy with reconstruction and closure of previous tracheostoma. Median duration until decannulation for the planned tracheostomy patients was 24.5 days (*n* = 6, range: 11–127 days, mean 50 days). Thus, tracheotomy dependency is low with 85.7% decannulation rate (*n* = 6/7) after PDT treatment. One patient was never decannulated due to progressive disease.

The most expected local adverse event of tumor site necrosis (Figure 1) occurred in 88.5% (*n* = 23) of cases. In one of three cases without necrosis, progressive disease was present. In the other two cases complete tumor response was observed. We observed that local healing was complete at a median local healing time of 107 days (3–4 months; mean 143, *n* = 21, range: 30–604 days). In 5 patients failure of observing full healing was due to tumor recurrence in 4 and to a tumor-unrelated death (acute CVA) in the early follow-up period. Out of the four patients with recurrence, according to the RECIST tumor response criteria (15), two presented stable disease and the other two progressive disease at a median follow-up time of 57 days (*n* = 4, range 22–85 days, mean 55 days). Other local adverse effects in order of prevalence were: facial edema (*n* = 13, 50%), nasal regurgitation (*n* = 9, 34.6%), injection site reaction (*n* = 7, 26.9%), spontaneous burns of various degrees (*n* = 7, 26.9%), trismus (*n* = 7, 26.9%), phlebitis (*n* = 5, 19.2%), necrotizing stomatitis (*n* = 5.19.2%), oronasal fistula (*n* = 1, 3.8%) (**Figure 5**) and velopharyngeal insufficiency (*n* = 1, 3.8%). Systemic incidental indirect adverse events during hospital stay illustrate the frailty of this patient population and the impact of PDT treatment in this instance: delirium, takotsubo cardiomyopathy, cardiac arrhythmia, urinary retention, aspiration pneumonia, acute cerebrovascular accident (CVA).

## Discussion

We studied 26 patients with oral and/or oropharyngeal cancer treated with photodynamic therapy (PDT). Complete tumor response occurred in 20 out of 26 patients (76.9%). Partial response was noted in three cases (11.5%) and progressive disease was observed in the remaining three cases (11.5%). Tan et al. (4) in a multicenter study of 39 patients, report a complete response in 49% of cases. In our study, local control was obtained in 42.3% (*n* = 11) of the patients. In case of recurrence (*n* = 21), almost two thirds (*n* = 5, 71.4%) of the tumors appeared at the same or adjacent site as the illuminated tumor. A possible explanation is field cancerization, which involves the expansion and migration of clonally related preneoplastic cells (16), first described by Slaughter et al. (17) who found histologically altered tissue surrounding squamous cell carcinoma.

In our case series mTHPC (Foscan) was used as the photosensitizing agent, as it is the most potent second generation photosensitizing agent currently available and approved in Europe since 2001 for the palliative use in squamous cell carcinoma of the head and neck (18). Newer alternative photosensitizers, with a more favorable photophysical and pharmacokinetic profile, are in development due to need of prolonged protection from sunlight and other sources of bright light after PDT treatment with mTHPC (19). However, further studies with larger patient populations and longer follow-up are needed to confirm the clinical efficacy of these newer agents. Targeted delivery methods of photosensitizers with various nanoparticles as a carrier system, are also being explored, hopefully further expanding the application of PDT for thick and bulky HNSCC without the need for interstitial PDT (20). In the present study, no significant difference in survival and functional outcome was observed between superficial PDT in comparison to interstitial or combined PDT.

Twenty-one of these patients (80.8%) had a history of combined smoking and alcohol abuse. A synergistic effect is proven of smoking and alcohol on the risk of oral cancer (21, 22). Four patients (15.4%) continued to combine smoking and alcohol consumption after receiving PDT. The higher number of isolated alcohol consumption (*n* = 9, 34.6%) post-PDT is most likely due to the fact that part of the combination abusers gave up smoking but were not able to stop alcohol consumption entirely. It is no surprise that all but one of these patients had an extensive oncological history in head and neck squamous cell carcinoma (HNSCC) at various sites (Table 2). All were histologically typed as squamous cell carcinoma (SCC) to a variable degree of differentiation, with one verrucous carcinoma. This is in line with the overall percentage of SCC, which accounts for more than 90% of all head and neck cancers (21). Only two patients had a definite negative p16 status, one in each subgroup. The p16 status of the tumors in this study was not systematically assessed, since the cohort spans a broad time-line, dating back to January 2002, before routine p16 staining and HPV *in situ* hybridization was introduced in our center.

The conventional treatment modalities of HNSCC in the curative setting are primary radio(chemo)therapy, ablative surgery alone, or a combination of both treatments (6). In this retrospective study, 15 patients (57.7%) were treated with a combination of radio(chemo)therapy and ablative surgery prior to illumination. Only one patient received PDT as a first treatment modality, and had not received any prior tumor management. Ablative surgery with broad margins is usually preferred due to higher survival rates. On the other hand, surgery has the disadvantage of being mutilating at times, and depending on the localization, size, and distribution of the tumor, the patient can be functionally inoperable or even oncologically unresectable. When performing PDT however, tumor margins need to be taken in account as well. In the multicenter study by D'cruz et al. (8) a minimal margin of 5 mm was used. Likewise, we also implemented a minimal margin of 5–10 mm healthy tissue margin whenever anatomically possible.

Common morbidity after head and neck oncological surgery for HNSCC is observed in swallowing (dysphagia), loss of speech and/or articulation, unfavorable cosmesis, etc. Nonetheless, radiotherapy also has some common non-negligible complications such as: xerostomia, dysgeusia, dysphagia, and osteoradionecrosis (6).

About one third of our patients (*n* = 9, 34.6%) developed a separate primary tumor outside of the head and neck region in their follow-up period after PDT. Notably, the sites of these new primary tumors are also notoriously associated with alcohol abuse and smoking: esophageal cancer, lung cancer, breast cancer, and bladder cancer. Other separate primary tumors were prostate cancer and sarcoma (23–25).

**Figure 4:**
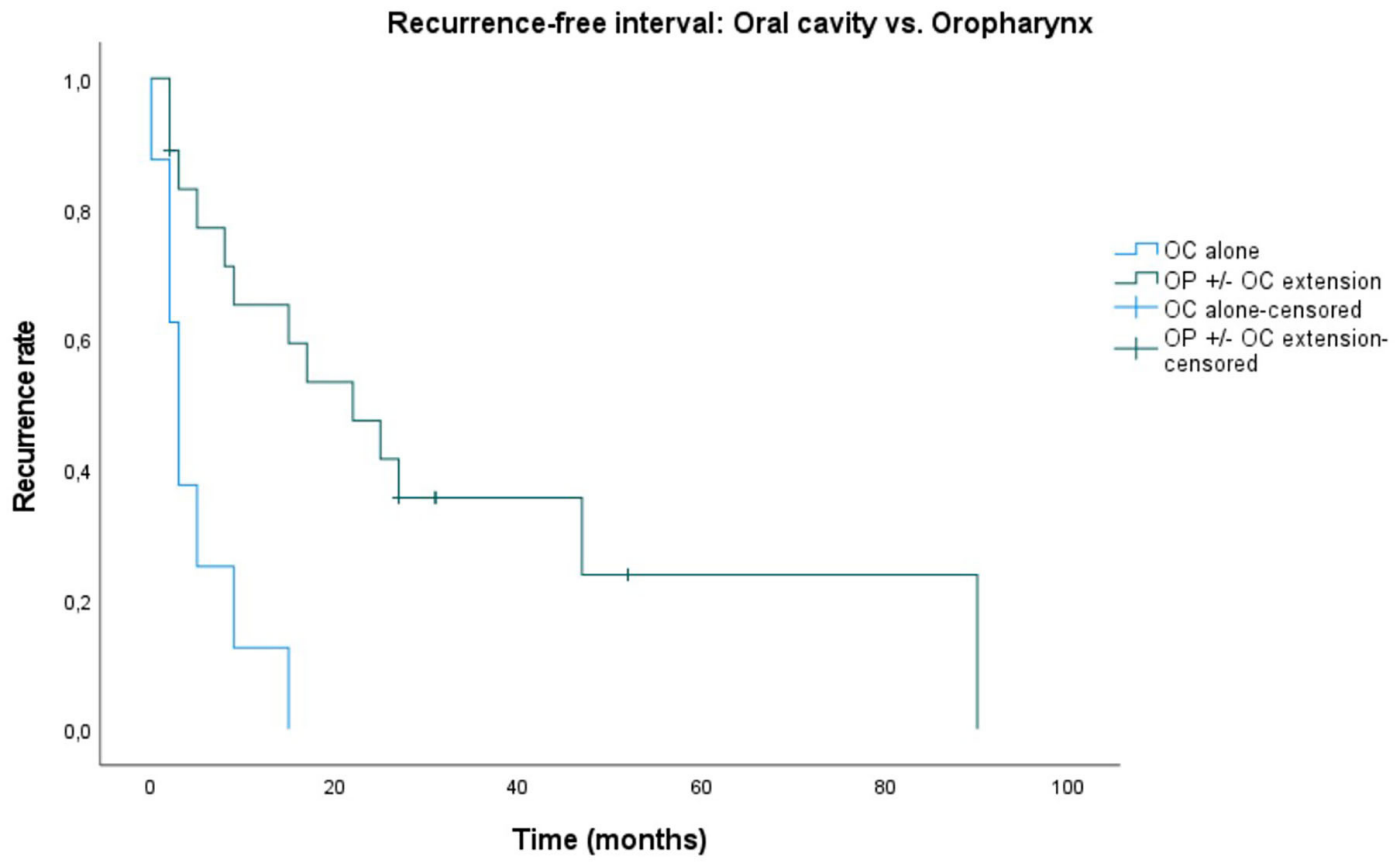
Kaplan–Meier plot showing statistically significant difference in recurrence-free interval (*p* < 0.001) for oropharyngeal squamous cell carcinoma (with or without oral cavity extension) treated with photodynamic therapy, compared to oral cavity tumors alone. Starting point in time is PDT illumination date until local recurrence. OC, oral cavity; OP ± OC extension, oropharyngeal squamous cell carcinoma with or without oral cavity extension.

Median overall survival in the systematic review of by De Visscher et al. (12) was 8–16 months. In our patient population, median overall survival (OS) was 31 months (2–129 months). We created Kaplan–Meier plots for OS, and DSS (Figures 2, 3, respectively) and reported the median outcome 6 months, 1, 2, and 5 years after PDT. We looked at different variables including gender, age, TNM classification, substance abuse, and number of recurrences for which treatment with PDT. No statistically significant factor with an impact on OS or DSS was identified. This is most likely at least in part due to the small sample size and the rarity of this treatment modality as well as its indication, which defines a selection of patients with superficial tumors without lymph node involvement or distant metastasis. Nevertheless, the long median survival is remarkable. Particularly in oropharyngeal SCC with or without oral cavity extension, we observed a durable local control. These patients were found to have a significantly better recurrence-free interval (*p* < 0.001) compared to oral cavity SCC alone (Figure 4). This difference in outcome could be due to a fraction of oropharyngeal SCC being HPV-induced, unfortunately the lack of consistent HPV/p16 data prevents us from substantiating this possibility. On the other hand, it is not unlikely that most of these oropharyngeal cancers were not HPV related: most (*n* = 10, 71.4%) were soft palate tumors and all patients had a strong tobacco and alcohol past. The rate of soft palate tumors in oropharyngeal cases is comparable to the literature, showing a 75% occurrence in the oropharyngeal tumors analyzed in the systematic review by De Visscher et al. (12). We presume that a more favorable exposure for PDT illumination may also have played a role. Finally, more than half (*n* = 11, 61%) of the oropharyngeal tumors were new primary lesions, whereas tumors in the oral cavity subgroup were more often recurrences (*n* = 6, 75%). The higher rate of new primary lesions in the oropharynx subgroup might contribute to help explain the significant difference in recurrent free interval. Although a significant correlation was not observed in our case series, De Visscher et al. (12) showed a 83% complete tumor response rate for first primary tumors as opposed to 67% in non-primary tumors (*p* = 0.001). There is a paucity of data available in regards to HPV status in PDT studies. Many noteworthy reviews (5, 21), case series (4, 19), and systematic reviews (6, 12) make no comment on it. Interestingly, a recent *in vitro* research study on HPV related sensitivity toward radiation and PDT showed an unexpected but significant difference in sensitivity patterns: Kessel et al. (26) found that a cell line derived from a donor with a HPV infection was more responsive to radiation, but significantly less responsive to PDT than a cell line derived from an HPV-free patient. The authors of this study cannot postulate a simple explanation for this finding, as they observed no impaired photosensitizer uptake or decreased reactive oxygen species formation in the HPV positive cell line. The primary goal of this study was to examine the responsiveness of HPV-negative cells to PDT through paraptosis. Their research shows morphologic evidence for paraptosis after PDT to HPV negative cells, and a significantly less responsive effect from radiotherapy in comparison with PDT due to an impaired apoptosis pathway (26).

Further *in vitro* studies, as well as clinical data, is necessary in order to better understand the effect on PDT in relation to the HPV status of the tumor.

Besides the oncological outcome, we also studied adverse events and toxicity of PDT. When evaluating PDT as a potential treatment modality, a thorough assessment of treatment toxicity on target organ functionality is paramount. Preserved swallowing function, defined as complete oral feeding with no supplementation, was observed at the end of follow-up for 76.9% of cases in our study. Upper airway functionality remained uncompromised in 95.7% of patients that did not have a pre-existing permanent tracheostoma at the end of follow-up. Thus, tracheotomy dependency is low with a 85.7% decannulation rate after PDT treatment with planned tracheotomy. Only one patient was never decannulated due to progressive disease. The decision to perform prophylactic tracheotomy was based on a case per case evaluation of tumor stage, tumor site, and anticipated difficult airway. In general, when planning PDT for a large tumor surface area located in the oropharyngeal region (base of tongue, soft palate, and posterior pharyngeal wall), a preventive tracheotomy was performed, especially in case of risk factors for a difficult airway, such as trismus, a history of radiation or extensive surgery to the head and neck.

**Figure 5:**
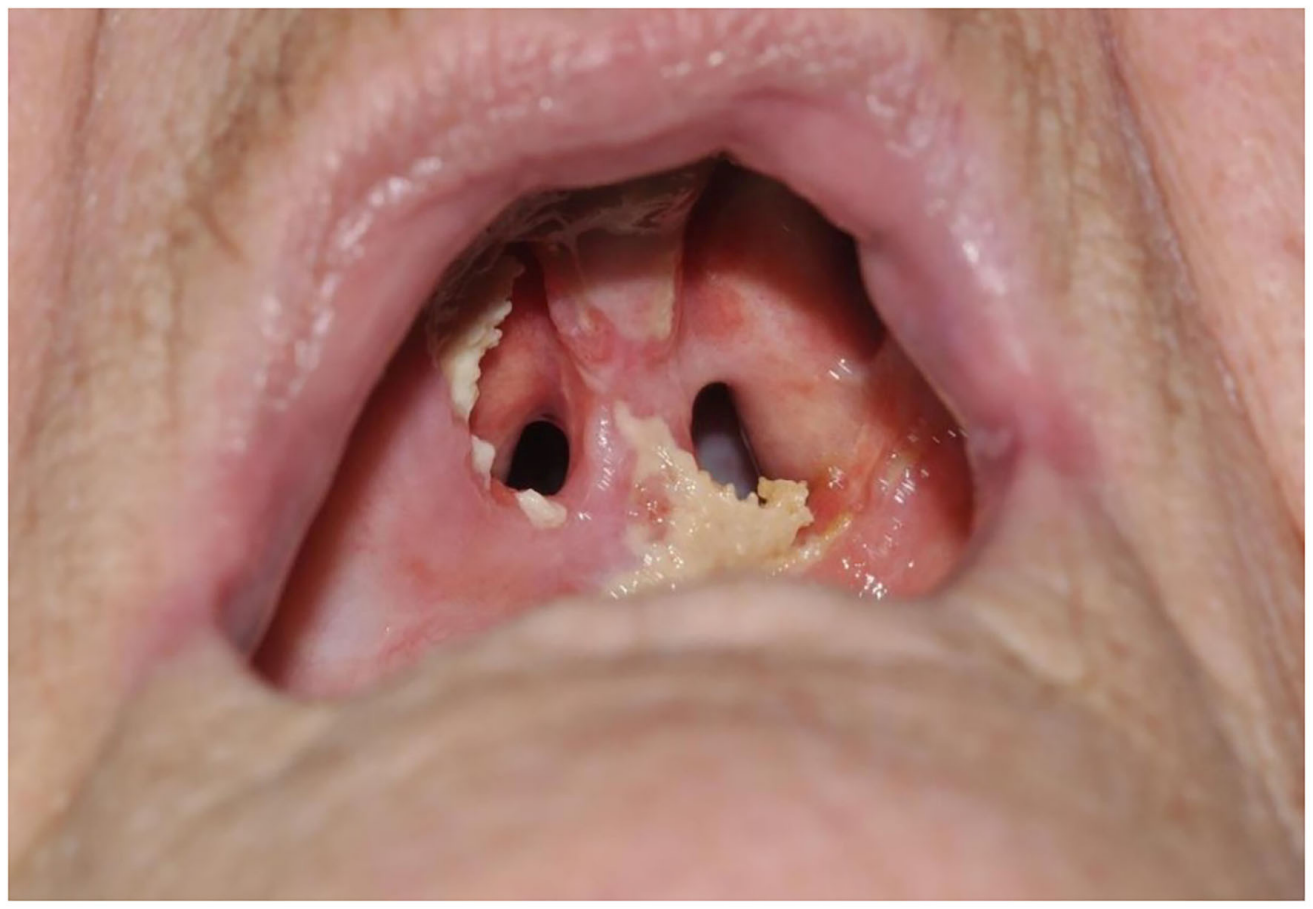
Photodynamic therapy patient with oronasal fistula, 3 months after PDT illumination of the hard palate.

In first instance, out of all adverse events, pain and dysphagia are to be expected. Secondly, facial edema, injection site reactions, burns, nasal regurgitation, trismus, and phlebitis are not uncommon. Tumor necrosis can explain pain after treatment and facial edema. Extensive necrosis can cause exposed bone and oronasal fistula (Figure 5) and even cerebrospinal fluid leakage in case of PDT on the skull base. In our study, complete local healing was observed at a median time of 107 days or 3–4 months healing time. Four patients recurred before complete local healing could occur at a median follow-up time of 57 days. In other words, when no progressive healing or rather suspicious tissue is observed after ~2 months, tumor progression or early recurrence needs to be considered and a low threshold for biopsy is warranted. Phlebitis and injection site reactions are caused by intravenous administration of mTHPC. Burns occur due to the phototoxic effects of mTHPC. Trismus, dysphagia and nasal regurgitation are possible complications because of tissue scarring and the development of fistula after PDT. Delirium, arrhythmia, and cardiomyopathy might be explained by intrinsic factors of the patient population, namely a frail elderly population with a history of smoking and alcohol abuse and stress caused by hospitalization. The adverse events due to photodynamic therapy are mostly mild, but in one case laryngeal edema caused an upper airway obstruction. Adverse events reported in the literature are largely similar to those in our patient population: injection site reactions (11%), edema (11%) (8), trismus (8%), necrotizing stomatitis (5%), vomiting (5%), and dysphagia(13%) (4). Local pain, pruritus (5), burns, orocutaneous fistula, skin necrosis, and acute airway obstruction (7) have also been described. Burns can be avoided if the patient stays inside, away from bright light (6). In the post-PDT illumination period, our patients are instructed on the safety measures regarding light and sun exposure. A commercially available LUX-meter is provided for each patient, which is used during the hospital admission and at home after discharge, to monitor light exposure. The PDT patients are instructed to progressively increase the light exposure by no more than 100 Lux each day. In addition, every patient receives a brochure with a timeline on progressive light exposure and the necessary information on preventive measurements such as clothing and the avoidance of certain light-emitting appliances (computer, smart phone screens, etc.). In case of burns secondary to the PDT treatment a careful evaluation and follow-up by a specialized burn unit is essential, as second degree burns due to accidental light exposure is not uncommon. During the hospital admission local and systemic effects are monitored, pain is controlled with paracetamol, NSAID and/or opioid pain medication. Usually a prolonged course of 1.5 g metronidazole was administered in three divided daily doses for several weeks to prevent local bacterial colonization of the necrotic tumor site. Evidently, regular debridement of the necrotic tissue is indicated to further reduce the risk of surinfection and promote mucosal healing. Corticosteroid administration is ideally reserved for cases of manifest edema and possible airway risk, as it may inhibit the potential systemic antitumor immunological reaction.

Limitations of this study are the relatively small number of patients treated with PDT over a long period of time, the lack of quality of life measurements, the retrospective study design, and the fact that this study does not compare PDT to standard treatment. Quality of life increase in PDT patients with HNSCC has been demonstrated in the past (4, 8, 12), however, comparative studies to conventional treatment options are currently still lacking. New prospective trials should aim to conduct systematic quality of life assessments before, during, and after PDT therapy to better illustrate the added value of this treatment modality. Due to the long time interval of inclusion, and the inherent long history of HNSCC in many of these patients, the 7th TNM classification system was mostly implemented at the time of diagnosis. Similarly, p16 staining and HPV *in situ* hybridization was not yet routine practice. The strong prevalence of substance abuse in this population as well as the development of multiple different sites of HNSCC, suggests these tumors to be clinically p16 negative. Still, the lack of p16 staining and unknown HPV status is a limitation in this study concerning the tumor biology. There is an overall paucity on data and research on the effects of PDT in HPV positive HNSCC, further *in vitro* research and clinical studies are necessary in order to determine a possible difference in tumor response. Strong points of this analysis are the consistency of treatment protocol and single treating physician, which has remained unchanged over the entire treatment period. Few studies comparing outcomes of PDT to surgical treatment have been published, with mainly positive results (10, 27). However, survival rates should not be compared to those of primary surgical cases, as these tumors treated with PDT are highly selected patients with most often recurrences of previously failed management. More importantly, toxicity and preserved organ function need to be taken into account when comparing PDT to other treatments. To date, there is not much data to support PDT as a primary treatment modality for invasive SCC. Still, a recent systematic review of Vohra et al. (28) showed that PDT is effective in the overall management of oral premalignant lesions.

To further prove the value of PDT in clinical practice, future prospective and controlled randomized studies with a specific treatment protocol and systematic quality of life measurements before, during, and after PDT should be carried out, comparing these outcomes to conventional therapy (6).

## Conclusion

The oncological outcome in this retrospective study is comparable to what has been previously reported in the literature. A complete response was obtained in 76% of the patients, with local control in 42.3%. Median overall survival was 24 months. The main cause of death was head and neck cancer (65%). Multiple but transient adverse events were reported in our study, mostly PDT specific.

In summary, PDT is a valuable treatment option in selected patients with oral and/or oropharyngeal HNSCC that induces durable local control in an important fraction of treated patients. The technique has an acceptable toxicity profile.

## Data Availability Statement

The datasets generated for this article are not readily available because the raw data supporting the conclusions of this article will be made available by the authors upon reasonable request. Requests to access the datasets should be directed to VV, vincent.vanderpoorten@uzleuven.be.

## Ethics Statement

The studies involving human participants were reviewed and approved by Research Ethics Committee UZ/KU Leuven. Written informed consent for participation was not required for this study in accordance with the national legislation and the institutional requirements.

## Author Contributions

AL: data quality control, data analysis (statistics), drafting manuscript, and review of manuscript. LN: data collection, data analysis (statistics), and drafting manuscript. SN, PC, JM, and PD: drafting manuscript and review of manuscript. VV: study concept, data quality control, data analysis (statistics), drafting manuscript, and review of manuscript. All authors contributed to the article and approved the submitted version.

## Conflict of Interest

The authors declare that the research was conducted in the absence of any commercial or financial relationships that could be construed as a potential conflict of interest.
